# Peripheral immunity involvement in the cognitive impairment of sporadic amyotrophic lateral sclerosis

**DOI:** 10.3389/fneur.2024.1405275

**Published:** 2024-05-31

**Authors:** Tianmi Yang, Qianqian Wei, Chunyu Li, Ruwei Ou, Junyu Lin, Yangfan Cheng, Yi Xiao, Huifang Shang

**Affiliations:** Department of Neurology, Laboratory of Neurodegenerative Disorders, Rare Diseases Center, National Clinical Research Center for Geriatrics, West China Hospital, Sichuan University, Chengdu, Sichuan, China

**Keywords:** amyotrophic lateral sclerosis, cognitive impairment, peripheral immunity, ALS, neutrophil, CD4+ T cell

## Abstract

**Background:**

Recent research has indicated the significance of immune activation in amyotrophic lateral sclerosis (ALS). However, the impact of peripheral immunity on cognitive impairment in sporadic ALS remains poorly characterized. Therefore, we aim to assess the relationship between peripheral immune parameters and cognitive impairment in patients with sporadic ALS.

**Methods:**

A case–control study involving 289 patients with sporadic ALS was conducted. All participants underwent cognitive assessment and measurements of blood immune parameters. The main outcomes included adjusted odds ratios (ORs) in multivariate logistic regression analysis and adjusted coefficients in a multivariate linear regression model. Sensitivity analysis was performed with stratification by the King’s clinical stage.

**Results:**

Cognitive impairment was observed in 98 (33.9%) patients. Higher counts of leukocyte (OR, 0.53; 95% CI, 0.29 to 0.95; *p* = 0.03), neutrophil (OR, 0.48; 95% CI, 0.26 to 0.88; *p* = 0.02), and monocyte (OR, 0.33; 95% CI, 0.18 to 0.60; *p* < 0.001) were significantly associated with better cognitive preformence in sporadic ALS, particularly among patients in King’s clinical stages 1 and 2. Conversely, a higher percentage of CD4+ T cells was linked to an increased risk of cognitive impairment (OR, 2.79; 95% CI, 1.52 to 5.09; *p* = 0.001), particularly evident in patients in King’s clinical stage 3.

**Conclusion:**

These results highlight the involvement of peripheral immunity in the cognitive impairment of sporadic ALS and suggest dynamic and intricate roles that vary across disease stages. Elucidating the links between immunity and ALS sheds light on the pathophysiological mechanisms underlying this fatal neurodegenerative disorder and informs potential immunotherapeutic strategies.

## Introduction

Amyotrophic lateral sclerosis (ALS) is a fatal neurodegenerative disease with significant socioeconomic and public health implications. In addition to progressive paralysis and weakness, cognitive impairment has gained increasing attention in research (1–4). Approximately 30–50% of ALS patients experience varying degrees of cognitive or behavioral impairment, with 5–15% also co-existing with frontotemporal dementia (FTD) (1, 2, 4). Previous cohort studies have demonstrated that cognitive impairment in ALS is associated with poor survival (1, 5). However, the mechanisms underlying cognitive impairment in ALS remain largely unknown.

Research into alterations of immune and inflammatory responses in ALS is an active area, presenting a potential therapeutic target (6–8). Studies have uncovered abnormalities in peripheral blood cells (including leukocytes, CD4+ T cells, CD8+ T cells, natural killer T cells, Th17 cells, regulatory T cells, and monocytes) and immune proteins (such as immunoglobulins, complement factors, cytokines, and chemokines) in ALS patients or animal models (6). This evidence indicated that various components of the peripheral immune system might participate in ALS by influencing neuroinflammation. In our previous study comparing ALS patients with matched healthy controls, we observed peripheral immune activation in ALS and noted an inverse correlation between CD4+ T cell percentage and the revised ALS Functional Rating Scale (ALSFRS-R) score (9). Additionally, a prospective cohort study revealed correlations between changes in neutrophil and CD4+ T cell numbers over the course of ALS and disease progression (10).

Cumulatively, an expanding body of evidence implicates immune activation as part of systemic changes in ALS and contributes to disease progression. However, whether and how peripheral immunity is involved in cognitive impairment in ALS patients remains to be determined. Therefore, we aim to test the hypothesis that peripheral immunity is involved in cognitive impairment and plays varying roles according to disease stages in patients with sporadic ALS.

## Patients and methods

### Study participants

Patients included in the study met the El Escorial revised criteria (11) for definite and probable ALS and were diagnosed between January 2018 and December 2019 at the Tertiary Motor Neuron Disease Treatment Center, West China Hospital of Sichuan University. Exclusion criteria included (1) any family history of ALS or dementia; (2) presence of infection (total leukocyte count >10 × 10^9^/L); (3) severe dysarthria or hand weakness, educational level < 3 years, or refuse to complete the cognitive assessment due to other serious medical conditions; (4) patients with incomplete blood test data were also excluded. Ethics approval for the study was approved by the institutional ethics committee of Sichuan University. Informed written consent had been provided by all patients. The study followed the Strengthening the Reporting of Observational Studies in Epidemiology (STROBE) reporting guideline for case–control studies (Supplementary Table S1).

### Data collection and cognitive assessment

Demographic and clinical characteristics including age at diagnosis, sex, educational level, site of onset, and disease duration (time from onset to diagnosis), were recorded. The severity of the disease was assessed using the ALSFRS-R. Disease stages were determined based on the King’s clinical staging system, which characterized progressive events of functional involvement across four central nervous system regions: bulbar, upper limb, lower limb, and diaphragmatic (12). Specifically, Stage 1 marked symptom onset, indicated by functional impairment in one region due to weakness, wasting, spasticity, dysarthria, or dysphagia. Stage 2 represented functional involvement of a second region, while Stage 3 denoted functional involvement of a third region. Finally, Stage 4 indicated the need for gastrostomy and non-invasive ventilation (12).

The China version of Addenbrooke’s Cognitive Examination–Revised (ACE-R) was utilized to evaluate cognitive function upon enrollment (13, 14). Lower ACE-R scores indicate poorer cognitive performance. Cognitive impairment was defined as a total ACE-R score < 75, a cutoff point established in our ALS cohort, which was 1.5 standard deviations (SD) below the mean value for healthy controls (14). Patients were categorized into two groups: ALS with cognitive impairment (ALS-ci) and ALS without cognitive impairment (ALS-nci), based on the defined cutoff value.

Additionally, the Hamilton Depression Scale (HAMD) and the Hamilton Anxiety Scale (HAMA) were utilized to assess depression and anxiety, with cutoff scores of ≤7 for both scales (15). Other potential factors, including smoking, drinking, and Riluzole usage, were also recorded.

### Blood immune parameter measurement

Peripheral blood samples were collected via venipuncture into tubes containing a clot activator and double gel (BD Vacutainer, SST, REF) between 8:00 and 11:00 am after an overnight fast in each patient. A complete blood count, including leukocyte, neutrophil, lymphocyte, monocyte, eosinophil, and basophil counts, was performed using an automatic blood cell analyzer. Lymphocyte subsets were identified and quantified using the BD FACSCanto II Flow Cytometer (Becton Dickinson Biosciences, Heidelberg, Germany). For each sample, at least 10,000 cells were analyzed, and the percentage of cells expressing CD3+, CD4+, and CD8+ markers was determined. Immunoglobulin levels (IgG, IgM, and IgA) and the concentrations of complement factors (C3, C4, and factor B) were quantified using nephelometry on an Image 800 nephelometer (Beckman Coulter, Fullerton, CA, United States). All experiments in this study were conducted in the Department of Laboratory Medicine at West China Hospital.

### Statistical analysis

Statistical analyses were conducted using SPSS version 26.0 (SPSS, Chicago, IL, United States) and GraphPad Prism version 9.0 (GraphPad Software, LLC.). The Shapiro–Wilk test was employed to assess the normality of distribution for each variable. Continuous variables are presented as mean (SD), while categorical variables are reported as numbers. Continuous variables were analyzed using either a Student’s t-test or ANOVA test, while categorical variables were assessed using χ^2^ tests. The Mann–Whitney U test was applied for variables with non-normal distribution. Statistical significance was defined as two-sided *p* values <0.05.

We evaluated the residuals to confirm they met all linear regression assumptions. Associations between immune parameters and cognition (ACE-R score) were first analyzed using univariate linear regression. Subsequently, these associations were further explored through multivariate linear regression models and multivariate logistic regression analysis, adjusting for age, sex, educational level, site of onset, disease duration, and ALSFRS-R scores. Furthermore, potential confounding variables, such as depression, anxiety, smoking, drinking, and the use of Riluzole, were also adjusted for. Binary variables for immune parameters were defined based on their mean values. Sensitivity analyses were conducted to determine if the impact of peripheral immunity on cognitive function remained consistent across various stages of ALS.

## Results

### Demographic and clinical characteristics

**Figure 1:**
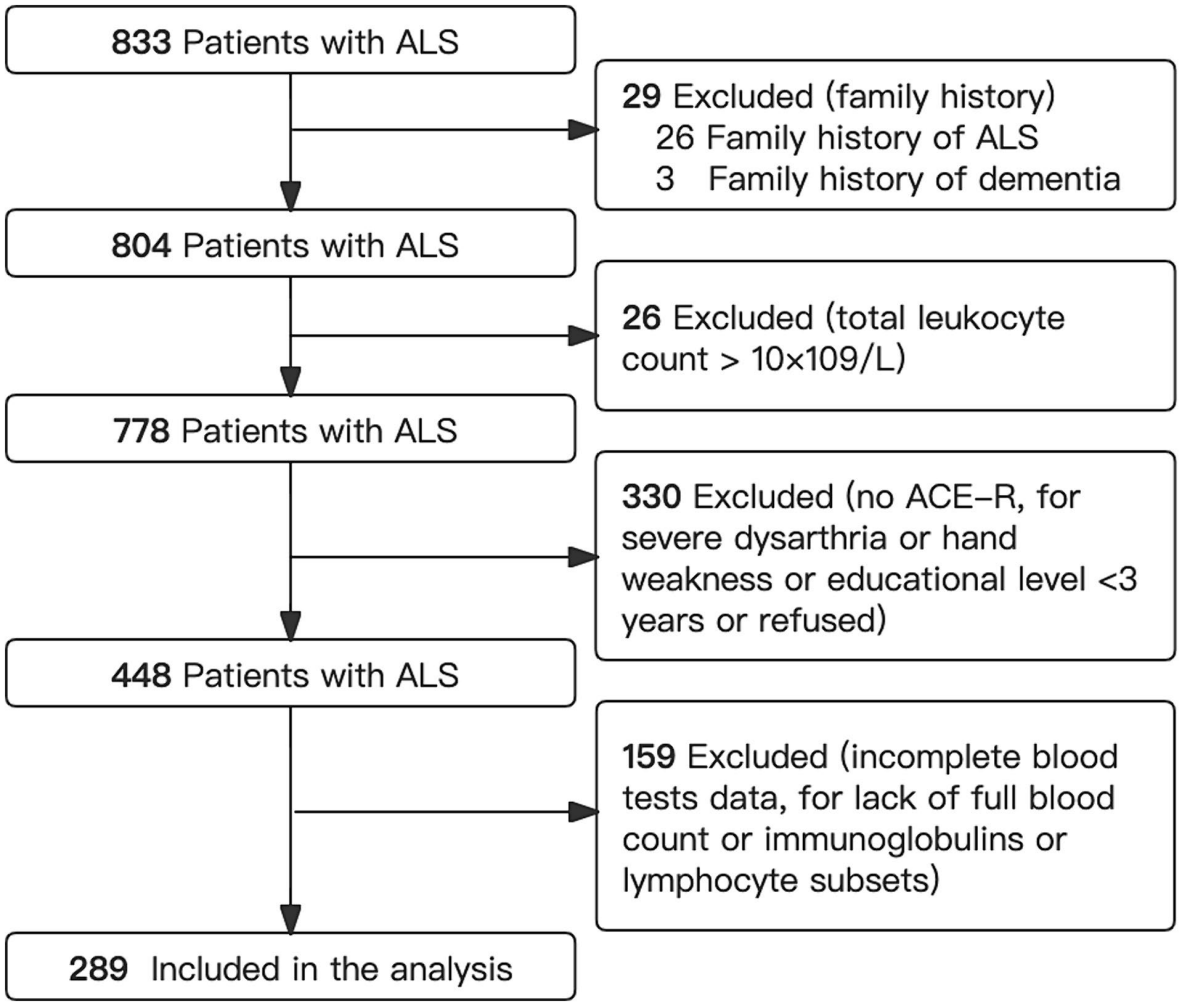
Flowchart showing selection of patients. ACE-R, Addenbrooke’s Cognitive Examination–Revised; ALS, amyotrophic lateral sclerosis.

With selection, a total of 289 eligible ALS patients with complete cognitive and immune parameters were included in our analysis (Figure 1). The mean age at diagnosis was 54.4 (11.7) years, with 186 (64.4%) being male (Table 1). The average disease duration for ALS was 14.7 (13.4) months, and the mean ALSFRS-R score was 41.1 (4.7). Among these patients, 123 (42.6%) were classified as stage 1, 93 (32.2%) as stage 2, 71 (24.6%) as stage 3, and only 2 (0.7%) as stage 4.

**Table 1:** Characteristics of patients with sporadic ALS with and without cognitive impairment.

Characteristic	Total (*N* = 289)	ALS-nci (*n* = 191)	ALS-ci (*n* = 98)	*p* value^a^
Age, mean (SD), y	54.4 (11.7)	51.8 (12.4)	59.4 (8.4)	< 0.001
Sex, Female/Male	103/186	63/128	40/58	0.19
Education level, y	9.2 (3.5)	10.3 (3.4)	7.1 (2.7)	< 0.001
Site of onset, Bulbar/Limb	37/252	22/169	15/83	0.36
Duration, month	14.7 (13.4)	14.0 (14.0)	16.1 (12.2)	0.22
ALSFRS-R, mean (SD) [range]	41.1 (4.7) [24–47]	41.5 (4.3) [24–47]	40.4 (5.2) [24–47]	0.05
ACE-R score, mean (SD) [range]	78.7 (13.0) [40–100]	86.4 (6.7) [75–100]	63.7 (8.4) [40–74]	< 0.001
HAMD, A/T	108/237	69/149	39/88	0.22
HAMA, A/T	70/240	45/151	25/89	0.55
Smoking, No. (%)	109 (37.7)	70 (36.6)	39 (39.8)	0.60
Drinking, No. (%)	75 (26.0)	49 (25.7)	26 (26.5)	0.87
Riluzole usage, No. (%)	121 (41.9)	80 (41.9)	41 (41.8)	0.99
Leukocyte, ×10^9^/L	6.20 (1.81)	6.47 (1.91)	5.68 (1.48)	< 0.001
Neutrophil, ×10^9^/L	3.76 (1.46)	3.97 (1.54)	3.36 (1.18)	0.001
Lymphocyte, ×10^9^/L	1.86 (0.61)	1.91 (0.62)	1.76 (0.57)	0.05
Monocyte, ×10^9^/L	0.40 (0.15)	0.42 (0.16)	0.36 (0.11)	0.001
Eosinophil, ×10^9^/L	0.15 (0.14)	0.14 (0.12)	0.17 (0.18)	0.09
Basophil, ×10^9^/L	0.03 (0.02)	0.03 (0.02)	0.03 (0.02)	0.09
CD3+ T cell, %	68.54 (9.64)	68.56 (9.83)	68.50 (9.30)	0.96
CD4+ T cell, %	39.54 (8.91)	38.75 (8.67)	41.09 (9.21)	0.03
CD8+ T cell, %	24.19 (7.80)	24.71 (7.69)	23.18 (7.95)	0.12
IgG, g/L	11.78 (2.44)	11.53 (2.29)	12.29 (2.64)	0.01
IgA, g/L	2.12 (0.87)	2.20 (0.93)	2.03 (0.74)	0.10
IgM, g/L	1.27 (0.71)	1.18 (0.54)	1.44 (0.93)	0.003
C3, g/L	0.86 (0.16)	0.88 (0.16)	0.83 (0.15)	0.01
C4, g/L	0.22 (0.06)	0.22 (0.06)	0.21 (0.06)	0.34
Factor B, g/L	0.31 (0.07)	0.32 (0.07)	0.30 (0.08)	0.12

ALS, amyotrophic lateral sclerosis; ALS-nci, ALS without cognitive impairment; ALS-ci, ALS with cognitive impairment; ALSFRS-R, Revised-ALS Functional Rating Scale; ACE-R, Addenbrooke’s Cognitive Examination–Revised; A/T, abnormal/total; HAMD, Hamilton depression scale; HAMA, Hamilton anxiety scale.

a *p* values indicate differences between patients with and without cognitive impairment, and *p* less than 0.05 was considered statistically significant.

Cognitive performance varied from normal to significantly impaired, with a mean ACE-R score of 78.7 (13.0). Cognitive impairment was observed in 98 (33.9%) patients. The ALS-ci group exhibited an older age at diagnosis (59.4 vs. 51.8 years, *p* < 0.001), lower educational level (7.1 vs. 10.3 years, *p* < 0.001), lower counts of leukocytes (5.68 vs. 6.47 × 10^9/L, *p* < 0.001), neutrophils (3.36 vs. 3.97 × 10^9/L, *p* = 0.001), monocytes (0.36 vs. 0.42 × 10^9/L, *p* = 0.001), a higher percentage of CD4+ T cells (41.09 vs. 38.75%, *p* = 0.03), higher levels of IgG (12.29 vs. 11.53 g/L, *p* = 0.01), IgM (1.44 vs. 1.18 g/L, *p* = 0.003), and a lower level of C3 (0.83 vs. 0.88 g/L, *p* = 0.01) compared to the ALS-nci group (Table 1).

### Associations between peripheral immune parameter and cognitive impairment

**Figure 2:**
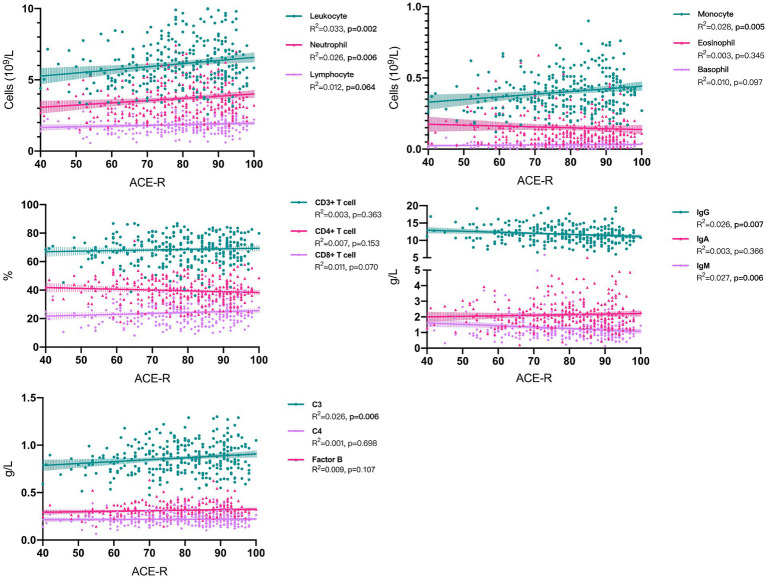
Associations between peripheral immune parameter and cognitive performance in sporadic ALS patients. ACE-R, Addenbrooke’s Cognitive Examination–Revised; ALS, amyotrophic lateral sclerosis.

In univariate linear regressions, higher counts of leukocytes (*p* = 0.002), neutrophils (*p* = 0.006), monocytes (*p* = 0.005), and a higher level of C3 (*p* = 0.006) were all significantly associated with better cognitive performance. Conversely, higher levels of IgG (*p* = 0.007) and IgM (*p* = 0.006) were negatively correlated with ACE-R scores (Figure 2 and Table 2). However, the lower R-square values suggested limited contributions of each immune parameter to cognitive changes, while the multivariate linear regression models demonstrated better fit and indicated the potential contributions of adjusted covariates to cognitive differences. With adjustment, we consistently observed significant positive correlations between leukocytes (*p* = 0.004), neutrophils (*p* = 0.02), monocytes (*p* = 0.001), and C3 (*p* = 0.02) with cognitive scores (Table 2).

**Table 2:** Univariate and multivariate linear regression analysis of peripheral immune parameter and cognitive performance (ACE-R scores) in sporadic ALS patients.

	Unadjusted				Adjusted^a^			
	Unstandardized β (SE)	Standardized β	*p*	*R* ^2^	Unstandardized β (SE)	Standardized β	*p*	*R* ^2^
Leukocyte, ×10^9^/L	1.497 (0.480)	0.181	0.002	0.033	1.140 (0.397)	0.138	0.004	0.368
Neutrophil, ×10^9^/L	1.695 (0.607)	0.163	0.006	0.026	1.228 (0.502)	0.118	0.02	0.363
Lymphocyte, ×10^9^/L	2.423 (1.250)	0.114	0.06	0.012	1.719 (1.026)	0.081	0.10	0.356
Monocyte, ×10^9^/L	14.866 (5.187)	0.167	0.005	0.028	14.480 (4.338)	0.162	0.001	0.374
Eosinophil, ×10^9^/L	−5.024 (5.311)	−0.056	0.35	0.003	−0.694 (4.337)	−0.008	0.87	0.35
Basophil, ×10^9^/L	60.595 (36.369)	0.098	0.10	0.01	43.417 (29.659)	0.07	0.14	0.354
CD3+ T cell, %	0.072 (0.079)	0.054	0.36	0.003	−0.018 (0.067)	−0.013	0.79	0.35
CD4+ T cell, %	−0.123 (0.086)	−0.084	0.15	0.007	−0.112 (0.07)	−0.077	0.11	0.355
CD8+ T cell, %	0.178 (0.098)	0.107	0.07	0.011	0.097 (0.080)	0.058	0.23	0.353
IgG, g/L	−0.851 (0.310)	−0.160	0.007	0.026	−0.508 (0.260)	−0.095	0.05	0.358
IgA, g/L	0.001 (0.001)	0.053	0.37	0.003	NA (0.001)	0	1.00	0.35
IgM, g/L	−0.003 (0.001)	−0.163	0.006	0.027	−0.001 (0.001)	−0.069	0.17	0.354
C3, g/L	13.173 (4.720)	0.163	0.006	0.026	9.242 (3.873)	0.114	0.02	0.362
C4, g/L	4.880 (12.575)	0.023	0.70	0.001	11.215 (10.216)	0.053	0.27	0.352
Factor B, g/L	0.017 (0.010)	0.095	0.11	0.009	0.015 (0.009)	0.087	0.07	0.357

ALS, amyotrophic lateral sclerosis; ACE-R, Addenbrooke’s Cognitive Examination–Revised; NA, not applicable.

a Multivariate linear regression analysis: adjusted for age, sex, educational level, site of onset, disease duration and ALSFRS-R scores.

Consistently, in the multivariate logistic regression analysis (Table 3), we observed that higher counts of leukocytes (OR, 0.53; 95% CI, 0.29 to 0.95; *p* = 0.03), neutrophils (OR, 0.48; 95% CI, 0.26 to 0.88; *p* = 0.02), and monocytes (OR, 0.33; 95% CI, 0.18 to 0.60; *p* < 0.001) were significantly associated with a decreased risk of cognitive impairment in ALS after adjusting for age, sex, educational level, site of onset, disease duration, and ALSFRS-R scores. Notably, a higher percentage of CD4+ T cells was found to increase the risk of cognitive impairment in ALS (OR, 2.79; 95% CI, 1.52 to 5.09; *p* = 0.001).

**Table 3:** Multivariate logistic regression and sensitivity analyses (restricted by the King’s clinical stage) of peripheral immune parameter and cognitive impairment in sporadic ALS patients.

	Total (*N* = 289)		Stage 1 (*n* = 123)		Stage 2 (*n* = 93)		Stage 3 (*n* = 71)	
	OR (95% CI)	*p* value^a^	OR (95% CI)	*p* value^a^	OR (95% CI)	*p* value^a^	OR (95% CI)	*p* value^a^
Leukocyte, ×10^9^/L	0.53 (0.29–0.95)	0.03	NA	0.98	0.29 (0.10–0.84)	0.02	NA	0.09
Neutrophil, ×10^9^/L	0.48 (0.26–0.88)	0.02	NA	0.58	0.27 (0.09–0.80)	0.02	NA	0.26
Lymphocyte, ×10^9^/L	NA	0.12	NA	0.31	NA	0.42	NA	0.26
Monocyte, ×10^9^/L	0.33 (0.18–0.60)	< 0.001	0.26 (0.10–0.72)	0.009	NA	0.08	NA	0.05
Eosinophil, ×10^9^/L	NA	0.12	NA	0.25	NA	0.46	12.57 (2.41–65.55)	0.003
Basophil, ×10^9^/L	NA	0.70	NA	0.31	0.32 (0.12–0.85)	0.02	5.94 (1.37–25.82)	0.02
CD3+ T cell, %	NA	0.21	NA	0.92	NA	0.41	NA	0.31
CD4+ T cell, %	2.79 (1.52–5.09)	0.001	NA	0.12	NA	0.10	5.53 (1.41–21.61)	0.01
CD8+ T cell, %	NA	0.26	NA	0.74	NA	0.85	NA	0.31
IgG, g/L	NA	0.16	NA	0.66	NA	0.58	NA	0.20
IgA, g/L	NA	0.53	NA	0.89	NA	0.44	NA	0.85
IgM, g/L	NA	0.59	NA	0.80	NA	0.75	NA	0.45
C3, g/L	NA	0.69	NA	0.23	NA	0.97	NA	0.84
C4, g/L	NA	0.19	NA	0.15	NA	0.40	NA	0.53
Factor B, g/L	0.43 (0.24–0.79)	0.006	NA	0.20	0.32 (0.11–0.94)	0.04	NA	0.20

ALS, amyotrophic lateral sclerosis; NA, not applicable.

a Multivariable logistic model: adjusted for age, sex, educational level, site of onset, disease duration and ALSFRS-R scores.

In the multivariate linear or logistic regression analyses (Tables 2, 3), we found no significant associations between the count of lymphocytes, eosinophils, and basophils, the percentage of CD3+ T cells, CD8+ T cells, the levels of IgG, IgM, IgA, the concentration of C4, and ACE-R score. In addition, further adjustment for depression, anxiety, smoking, drinking, and the use of Riluzole did not alter the main findings.

### Sensitivity analyses

Demographic and clinical data for different stages are presented in Supplementary Table S2. When we limited the analysis to patients with ALS in stage 1, stage 2, and stage 3 individually, the results were consistent with our main analysis. Notably, we observed varying dominant roles of different immune parameters across disease phases. For instance, a higher count of monocytes (OR, 0.26; 95% CI, 0.10 to 0.72; *p* = 0.009) remained a protective factor for cognitive impairment in stage 1, but this association weakened and became non-significant in stage 2 or 3. Similarly, elevated counts of leukocytes and neutrophils continued to show significant associations with improved cognition among patients in stage 2, as evidenced by both multivariate linear and logistic regression analyses (Table 3 and Supplementary Table S3). Conversely, an increased eosinophil count (*p* = 0.003) and percentage of CD4+ T cells (*p* = 0.01) were linked to a higher risk of cognitive impairment among patients in stage 3, although these associations were not statistically significant in stages 1 or 2. Interestingly, basophil levels showed a decreased risk of cognitive impairment in stage 2 but an elevated risk in stage 3 (Table 3).

## Discussion

The comprehensive case–control study with complete datasets investigated the relationships between various peripheral immune markers and cognitive impairment in sporadic ALS patients. The findings supported the involvement of peripheral immunity in cognitive impairment among ALS patients. Furthermore, sensitivity analyses revealed that peripheral immune alterations might exert varying effects at different stages of ALS.

Previous research has highlighted associations between peripheral immune cells such as neutrophils, leukocytes, and CD4+ T cells, and ALS risk or progression (6, 9, 10, 16–22). Large prospective cohort studies from the UB Biobank demonstrated a significant correlation between increased baseline levels of neutrophils and elevated ALS risk (22). Concurrently, some studies have indicated that the total number of neutrophils increases over time and positively correlates with disease progression in ALS patients (10). Other studies have suggested that the neutrophilic response is responsible for initiating the reparative process of damaged tissue (23) and contributes to neuronal repair (24). While, our analysis revealed a protective role of neutrophils in cognitive function among ALS patients. The inconsistency of these findings may be attributed to the timing of immune marker detection, which may have different effects on ALS before or after onset. Additionally, the influence of various confounding factors and the dynamic nature of immune markers cannot be overlooked.

Regarding the role of CD4+ T cells in ALS, findings have also been controversial: while some studies have shown a negative correlation between the number of CD4+ T cells and ALSFRS-R scores (9, 10, 16), others have suggested a neuroprotective role for CD4+ T cells, particularly CD4 + CD25+ regulatory T cells, in ALS patients (17). We found that an increase in CD4+ T cells was associated with an elevated risk of cognitive impairment, especially in ALS patients with advanced stages. To our knowledge, only one cross-sectional study has explored the relationship between peripheral blood lymphocyte subsets and cognitive status in ALS patients (25). In that study, the authors found that a higher count of CD4+ T cells decreased the risk of cognitive impairment, which contrasts with our findings. The relatively small sample size (n = 81) and limited statistical power may contribute to this discrepancy. Furthermore, the disease stages of the included patients were unknown based on the available information (25).

In a broader context, eosinophils and basophils are recognized for their roles in modulating inflammation during parasitic infections and allergic reactions. In the field of ALS research, limited studies have observed an increase in eosinophil counts among ALS patients compared to healthy controls, with this elevation being inversely correlated with disease progression, suggesting a protective effect against ALS (26). However, an elevation of eosinophil-derived neurotoxin in the serum of ALS patients would promote neuroinflammation and oxidative stress (27). Genome-wide association analysis and Mendelian randomization studies have linked higher levels of eosinophils with an earlier onset of ALS, indicating a potential harmful role of eosinophils in ALS pathophysiology (28). Similar associations with ALS have not been reported for basophils (21, 26–28). In our analysis, we found that elevated levels of both eosinophils and basophils in late-stage ALS patients were linked to an increased risk of cognitive impairment. Future research endeavors should delve deeper into the underlying mechanisms of these observations and elucidate the specific roles of eosinophils and basophils in ALS pathophysiology.

In the context of cognitive impairment, investigations into the correlation between immune dysregulation and neuroinflammation in dementia offers crucial insights into understanding the role of immunity in cognitive impairment in ALS. Prospective analyses from the UK Biobank have revealed that increased innate immunity markers, such as neutrophils and systemic immune-inflammatory index, were associated with higher dementia incidence, while increased adaptive immunity markers, including lymphocytes, were associated with lower dementia incidence (29). This supports a dual role of peripheral immune alterations in dementia (29). Mechanistic studies on the role of neutrophils in Alzheimer’s disease (AD) are relatively extensive. It has been proposed that in transgenic AD mouse models, neutrophils can adhere to and stagnate capillaries, leading to reduced cerebral blood flow (30). Furthermore, neutrophils have been observed to infiltrate the brain, secrete inflammatory cytokines, and interact with microglia, exacerbating blood–brain barrier damage, synaptic dysfunction, pathological protein deposition, and the worsening of memory decline (31). Early depletion or blockade of neutrophils can significantly improve cerebral blood flow, reduce neuropathology, and ameliorate memory deficits (30, 31). Additionally, in AD mouse models, depleting or pharmacologically inhibiting peripheral T regulatory cells has been shown to enhance Aβ plaque clearance, alleviate neuroinflammatory responses, and reverse cognitive decline (32). Returning to our topic, although mechanistic investigations into peripheral immunity and cognitive impairment in ALS are currently lacking, postmortem examinations have revealed that, besides TDP-43 pathology, neuroinflammatory features such as lymphocytic and macrophage infiltration, and extensive microglial activation are distinct pathological alterations in ALS (33). Similar neuroinflammatory changes have been observed in the brains or spinal cords of individuals carrying SOD1 mutations, C9orf72 repeat expansions, and sporadic ALS (34–36). Evidence from ALS animal models also supports abnormal immune activation and neuroinflammation in ALS pathogenesis and disease progression (37). Currently, the relationship between central nervous system inflammation and peripheral immune system dysregulation, as well as their causality in ALS, remains uncertain. Overall, it is hypothesized that in the ALS disease state, components and functions of the peripheral immune system (including immune cells and their secreted immunomodulatory factors) may undergo alterations, driving systemic inflammation and contributing to a chronically proinflammatory microenvironment in collaboration with immune dysregulation in the central nervous system via a compromised blood–brain barrier (38). Future researches may benefit from integrating assessments of immune cells and cytokines to elucidate the mechanisms by which peripheral immune influences cognitive impairment in ALS patients.

This study has several limitations. Firstly, its cross-sectional design hinders causal interpretation of results: both cognitive assessment and immune parameter measurement were conducted at diagnosis, precluding assessment of temporal sequence in these associations. Thus, prospective data are required to further evaluate this potential causal link. Secondly, ALS-ci and ALS-nci were categorized roughly based on ACE-R scores. Although its high sensitivity and specificity for dementia detection have been established previously (13, 14), it may not be specific enough for ALS to categorize cognitive function (39). Thirdly, exclusion of participants unable to perform neuropsychological tests, or those with incomplete immune data, may introduce selection bias. Fourthly, while patients with positive family history were excluded, we cannot guarantee exclusion of sporadic patients with pathogenic mutations of ALS causative genes (such as *C9orf72*, *TARDBP*, etc.). Lastly, despite adjusting for age in the multivariate analysis, we cannot overlook the potential influence of significant age differences between ALS-ci and ALS-nci on immune parameters and cognitive function. Therefore, in future studies, utilizing age-matched participants would be preferable.

## Conclusion

Our results support the involvement of peripheral immunity in the cognitive impairment observed in sporadic ALS patients. Specifically, monocytes, neutrophils, leukocytes, and CD4+ T cells demonstrate dynamic roles in ALS cognition across various disease stages. These findings offer a novel perspective to enhance understanding of the interplay between peripheral immunity and ALS, thereby shedding light on potential immunotherapeutic strategies for ALS.

## Data availability statement

The original contributions presented in the study are included in the article/Supplementary material, further inquiries can be directed to the corresponding author.

## Ethics statement

Ethics approval for the study was approved by the institutional ethics committee of Sichuan University. Informed written consent had been provided by all patients. The studies were conducted in accordance with the local legislation and institutional requirements. Written informed consent for participation in this study was provided by the participants’ legal guardians/next of kin.

## Author contributions

TY: Conceptualization, Data curation, Formal analysis, Funding acquisition, Investigation, Methodology, Project administration, Resources, Software, Supervision, Validation, Visualization, Writing – original draft, Writing – review & editing. QW: Data curation, Formal analysis, Funding acquisition, Investigation, Methodology, Software, Supervision, Writing – review & editing. CL: Conceptualization, Formal analysis, Investigation, Methodology, Software, Supervision, Writing – review & editing. RO: Data curation, Formal analysis, Investigation, Methodology, Software, Writing – review & editing. JL: Data curation, Formal analysis, Methodology, Writing – review & editing. YC: Data curation, Formal analysis, Methodology, Writing – review & editing. YX: Data curation, Formal analysis, Validation, Writing – review & editing. HS: Conceptualization, Funding acquisition, Investigation, Supervision, Writing – original draft, Writing – review & editing.
