# Supplementary material for: Peripheral immunity involvement in the cognitive impairment of sporadic amyotrophic lateral sclerosis
Source: Front Neurol. 2024 May 31;15:1405275. doi: 10.3389/fneur.2024.1405275 (PMC11176427; doi:10.3389/fneur.2024.1405275)
Supplement: Supplementary file 1 [file Table_1.DOCX]

**Supplementary-Table 1. STROBE checklist for cohort study.**

|  | Item No | Recommendation | Page No |
| --- | --- | --- | --- |
| **Title and abstract** | 1 | (*a*) Indicate the study’s design with a commonly used term in the title or the abstract | 1-3 |
|  |  | (*b*) Provide in the abstract an informative and balanced summary of what was done and what was found | 3 |
| Introduction | | | |
| Background/rationale | 2 | Explain the scientific background and rationale for the investigation being reported | 3-4 |
| Objectives | 3 | State specific objectives, including any prespecified hypotheses | 4 |
| Methods | | | |
| Study design | 4 | Present key elements of study design early in the paper | 5-6 |
| Setting | 5 | Describe the setting, locations, and relevant dates, including periods of recruitment, exposure, follow-up, and data collection | 5-6 |
| Participants | 6 | (*a*) Give the eligibility criteria, and the sources and methods of selection of participants. Describe methods of follow-up | 5-6 |
|  |  | (*b*) For matched studies, give matching criteria and number of exposed and unexposed | NA |
| Variables | 7 | Clearly define all outcomes, exposures, predictors, potential confounders, and effect modifiers. Give diagnostic criteria, if applicable | 6-7 |
| Data sources/ measurement | 8* | For each variable of interest, give sources of data and details of methods of assessment (measurement). Describe comparability of assessment methods if there is more than one group | 6-7 |
| Bias | 9 | Describe any efforts to address potential sources of bias | NA |
| Study size | 10 | Explain how the study size was arrived at | 5 |
| Quantitative variables | 11 | Explain how quantitative variables were handled in the analyses. If applicable, describe which groupings were chosen and why | 6-7 |
| Statistical methods | 12 | (*a*) Describe all statistical methods, including those used to control for confounding | 6-7 |
|  |  | (*b*) Describe any methods used to examine subgroups and interactions | 6-7 |
|  |  | (*c*) Explain how missing data were addressed | 7 |
|  |  | (*d*) If applicable, explain how loss to follow-up was addressed | NA |
|  |  | (*e*) Describe any sensitivity analyses | 7 |
| Results | | |  |
| Participants | 13* | (a) Report numbers of individuals at each stage of study—eg numbers potentially eligible, examined for eligibility, confirmed eligible, included in the study, completing follow-up, and analysed | 7-8 |
|  |  | (b) Give reasons for non-participation at each stage | NA |
|  |  | (c) Consider use of a flow diagram | NA |
| Descriptive data | 14* | (a) Give characteristics of study participants (eg demographic, clinical, social) and information on exposures and potential confounders | 8-9 |
|  |  | (b) Indicate number of participants with missing data for each variable of interest | 8 |
|  |  | (c) Summarise follow-up time (eg, average and total amount) | NA |
| Outcome data | 15* | Report numbers of outcome events or summary measures over time | NA |
| Main results | 16 | (*a*) Give unadjusted estimates and, if applicable, confounder-adjusted estimates and their precision (eg, 95% confidence interval). Make clear which confounders were adjusted for and why they were included | 7-9 |
|  |  | (*b*) Report category boundaries when continuous variables were categorized | 7-8 |
|  |  | (*c*) If relevant, consider translating estimates of relative risk into absolute risk for a meaningful time period | NA |
| Other analyses | 17 | Report other analyses done—eg analyses of subgroups and interactions, and sensitivity analyses | 9-10 |
| Discussion | | | |
| Key results | 18 | Summarise key results with reference to study objectives | 10-11 |
| Limitations | 19 | Discuss limitations of the study, taking into account sources of potential bias or imprecision. Discuss both direction and magnitude of any potential bias | 13 |
| Interpretation | 20 | Give a cautious overall interpretation of results considering objectives, limitations, multiplicity of analyses, results from similar studies, and other relevant evidence | 11-12 |
| Generalisability | 21 | Discuss the generalisability (external validity) of the study results | 11-12 |
| Other information | | | |
| Funding | 22 | Give the source of funding and the role of the funders for the present study and, if applicable, for the original study on which the present article is based | 15 |

**Supplementary-Table 2.** **Demographic, clinical and peripheral immune characteristics of sporadic ALS patients according to the King’s clinical stage**

| **Characteristic** | **Total (N=289)** | **Stage 1 (n=123)** | **Stage 2 (n=93)** | **Stage 3 (n=71)** | ***P* ^a^** |
| --- | --- | --- | --- | --- | --- |
| Age, mean (SD), y | 54.4 (11.7) | 54.4 (11.8) | 54.3 (11.7) | 54.2 (11.9) | 0.69 |
| Sex, Female/Male | 103/186 | 40/83 | 35/58 | 28/43 | 0.57 |
| Education level, y | 9.2 (3.5) | 9.7 (3.5) | 8.8 (3.4) | 9.1 (3.4) | 0.63 |
| Site of onset, Bulbar/Limb | 37/252 | 16/107 | 11/82 | 10/61 | 0.91 |
| Duration, month | 14.7 (13.4) | 13.3 (9.6) | 14.7 (16.1) | 17.5 (15.1) | 0.006 |
| ALSFRS-R score | 41.1 (4.7) | 44.1 (2.2) | 40.8 (3.3) | 36.4 (5.4) | < 0.001 |
| ACE-R score | 78.7 (13.0) | 79.7 (13.1) | 78.5 (12.3) | 77.0 (13.7) | 0.91 |
| Cognition, ALS-ci/ALS-nci | 98/191 | 38/85 | 34/59 | 26/45 | 0.60 |
| Leukocyte, ×10^9^/L | 6.11 (1.57) | 6.07 (1.49) | 6.11 (1.57) | 6.18 (1.74) | 0.92 |
| Neutrophil, ×10^9^/L | 3.68 (1.25) | 3.58 (1.16) | 3.68 (1.18) | 3.82 (1.46) | 0.01 |
| Lymphocyte, ×10^9^/L | 1.86 (0.61) | 1.91 (0.56) | 1.85 (0.63) | 1.78 (0.66) | 0.67 |
| Monocyte, ×10^9^/L | 0.40 (0.15) | 0.40 (0.14) | 0.41 (0.16) | 0.39 (0.14) | 0.43 |
| Eosinophil, ×10^9^/L | 0.15 (0.14) | 0.15 (0.11) | 0.15 (0.15) | 0.15 (0.19) | 0.36 |
| Basophil, ×10^9^/L | 0.03 (0.02) | 0.03 (0.02) | 0.03 (0.02) | 0.03 (0.02) | 0.50 |
| CD3+ T cell, % | 68.54 (9.64) | 68.28 (9.81) | 68.46 (9.74) | 69.12 (9.41) | 0.75 |
| CD4+ T cell, % | 39.54 (8.91) | 38.65 (8.57) | 39.74 (9.45) | 40.82 (8.74) | 0.31 |
| CD8+ T cell, % | 24.19 (7.80) | 24.47 (7.62) | 24.40 (8.05) | 23.48 (7.92) | 0.97 |
| IgG, g/L | 11.78 (2.44) | 11.92 (2.21) | 11.49 (2.80) | 11.89 (2.32) | 0.08 |
| IgA, g/L | 2.12 (0.87) | 2.17 (0.93) | 2.08 (0.89) | 2.29 (0.75) | 0.26 |
| IgM, g/L | 1.27 (0.71) | 1.27 (0.71) | 1.24 (0.67) | 1.32 (0.76) | 0.94 |
| C3, g/L | 0.86 (0.16) | 0.87 (0.15) | 0.87 (0.17) | 0.85 (0.15) | 0.26 |
| C4, g/L | 0.22 (0.06) | 0.23 (0.07) | 0.21 (0.05) | 0.22 (0.06) | 0.65 |
| Factor B, g/L | 0.31(0.07) | 0.32 (0.07) | 0.30 (0.06) | 0.32 (0.08) | 0.26 |

ALS, amyotrophic lateral sclerosis; ALSFRS-R, Revised-ALS Functional Rating Scale; ACE-R, Addenbrooke’s Cognitive Examination–Revised; ALS-nci, ALS without cognitive impairment; ALS-ci, ALS with cognitive impairment.

^a^ *P* indicate differences among patients in stage1,2, and 3, and *P* less than .05 was considered statistically significant.

**Supplementary-Table 3.** **Multivariate linear regression analysis of peripheral immune parameters and cognitive performance (ACE-R scores) in sporadic ALS patients according to the King’s clinical stage**

|  | **Stage 1 (n=123)** | | | |  | **Stage 2 (n=93)** | | | |  | **Stage 3 (n=71)** | | | |
| --- | --- | --- | --- | --- | --- | --- | --- | --- | --- | --- | --- | --- | --- | --- |
|  | **Unstandardized β (SE)** | **Standardized β** | ***P ^b^*** | **R^2^** |  | **Unstandardized β (SE)** | **Standardized β** | ***P ^b^*** | **R^2^** |  | **Unstandardized β (SE)** | **Standardized β** | ***P ^b^*** | **R^2^** |
| Leukocyte, ×10^9^/L | 0.268 (0.667) | 0.03 | 0.69 | 0.344 |  | 2.304 (0.658) | 0.295 | 0.001 | 0.414 |  | 0.987 (0.762) | 0.125 | 0.20 | 0.42 |
| Neutrophil, ×10^9^/L | -0.043 (0.859) | -0.004 | 0.96 | 0.343 |  | 3.320 (0.872) | 0.319 | <0.001 | 0.426 |  | 0.849 (0.903) | 0.091 | 0.35 | 0.413 |
| Lymphocyte, ×10^9^/L | 1.326 (1.755) | 0.057 | 0.45 | 0.346 |  | 1.333 (1.724) | 0.068 | 0.44 | 0.337 |  | 3.142 (2.034) | 0.152 | 0.13 | 0.426 |
| Monocyte, ×10^9^/L | 13.763 (6.955) | 0.148 | 0.05 | 0.364 |  | 14.270 (7.279) | 0.185 | 0.05 | 0.36 |  | 18.150 (9.619) | 0.18 | 0.06 | 0.435 |
| Eosinophil, ×10^9^/L | -4.099 (9.425) | -0.033 | 0.66 | 0.344 |  | 10.093 (7.259) | 0.122 | 0.17 | 0.346 |  | -8.859 (6.769) | -0.123 | 0.20 | 0.42 |
| Basophil, ×10^9^/L | 48.092 (41.245) | 0.087 | 0.25 | 0.35 |  | 158.631 (61.559) | 0.219 | 0.01 | 0.379 |  | -72.539 (64.720) | -0.106 | 0.27 | 0.416 |
| CD3+ T cell, % | 0.037 (0.105) | 0.027 | 0.73 | 0.344 |  | 0.001 (0.115) | 0 | 1.00 | 0.332 |  | -0.159 (0.143) | -0.109 | 0.27 | 0.416 |
| CD4+ T cell, % | -0.014 (0.116) | -0.009 | 0.91 | 0.343 |  | -0.112 (0.115) | -0.086 | 0.33 | 0.339 |  | -0.255 (0.150) | -0.163 | 0.09 | 0.43 |
| CD8+ T cell, % | 0.049 (0.135) | 0.028 | 0.72 | 0.344 |  | 0.084 (0.134) | 0.055 | 0.63 | 0.335 |  | 0.120 (0.168) | 0.069 | 0.48 | 0.409 |
| IgG, g/L | -0.403 (0.471) | -0.068 | 0.39 | 0.347 |  | -0.188 (0.393) | -0.043 | 0.63 | 0.334 |  | -1.179 (0.553) | -0.2 | 0.04 | 0.443 |
| IgA, g/L | 0.001 (0.001) | 0.04 | 0.60 | 0.344 |  | NA (0.001) | 0.003 | 0.97 | 0.332 |  | -0.001 (0.002) | -0.046 | 0.63 | 0.407 |
| IgM, g/L | -0.002 (0.001) | -0.091 | 0.26 | 0.35 |  | -0.002 (0.002) | -0.124 | 0.18 | 0.346 |  | 0.001 (0.002) | 0.032 | 0.77 | 0.406 |
| C3, g/L | 19.541 (6.315) | 0.229 | 0.002 | 0.392 |  | 3.561 (6.616) | 0.051 | 0.59 | 0.334 |  | 3.438 (9.088) | 0.039 | 0.71 | 0.406 |
| C4, g/L | 24.647 (15.110) | 0.123 | 0.11 | 0.357 |  | 0.042 (20.453) | 0 | 1.00 | 0.332 |  | -2.422 (21.550) | -0.011 | 0.91 | 0.405 |
| Factor B, g/L | 0.026 (0.014) | 0.143 | 0.06 | 0.363 |  | 0.017 (0.017) | 0.087 | 0.33 | 0.339 |  | 0.004 (0.016) | 0.026 | 0.79 | 0.406 |

ALS, amyotrophic lateral sclerosis; ACE-R, Addenbrooke’s Cognitive Examination–Revised; NA, not applicable.

^b^ Multivariable linear regression model: adjusted for age, sex, educational level.
